# Epigenetic Regulation of miRNA Expression in Malignant Mesothelioma: miRNAs as Biomarkers of Early Diagnosis and Therapy

**DOI:** 10.3389/fonc.2019.01293

**Published:** 2019-11-29

**Authors:** Marco Tomasetti, Simona Gaetani, Federica Monaco, Jiri Neuzil, Lory Santarelli

**Affiliations:** ^1^Section of Occupational Medicine, Department of Clinical and Molecular Sciences, Polytechnic University of Marche, Ancona, Italy; ^2^Mitochondria, Apoptosis and Cancer Research Group, School of Medical Science, Griffith University, Southport, QLD, Australia; ^3^Molecular Therapy Group, Institute of Biotechnology, Czech Academy of Sciences, Prague, Czechia

**Keywords:** malignant mesothelioma, epi-miRNAs, miR-126, epigenetic biomarkers, early diagnosis

## Abstract

Asbestos exposure leads to epigenetic and epigenomic modifications that, in association with ROS-induced DNA damage, contribute to cancer onset. Few miRNAs epigenetically regulated in MM have been described in literature; miR-126, however, is one of them, and its expression is regulated by epigenetic mechanisms. Asbestos exposure induces early changes in the miRNAs, which are reversibly expressed as protective species, and their inability to reverse reflects the inability of the cells to restore the physiological miRNA levels despite the cessation of carcinogen exposure. Changes in miRNA expression, which results from genetic/epigenetic changes during tumor formation and evolution, can be detected in fluids and used as cancer biomarkers. This article has reviewed the epigenetic mechanisms involved in miRNA expression in MM, focusing on their role as biomarkers of early diagnosis and therapeutic effects.

## Introduction

Malignant mesothelioma (MM) is an aggressive malignancy, and its origin is largely associated with exposure to asbestos (1). Furthermore, asbestos exposure also increases the risk of lung cancers and a number of non-malignant diseases including pleural plaques, pleural effusions, and asbestosis (2). As a xenobiotic substance, asbestos contributes to the alteration of the genetic and epigenetic landscape (3). DNA is wrapped around histones that protect and regulate the packed DNA. This structure, chromatin, can be condensed and “closed,” a state associated with transcriptional repression, or it can be “open,” a state which allows proteins to access the DNA and thus inducing gene transcription. The chromatin structure is regulated and controlled by various post-transcriptional modifications, identified as epigenetic changes, which are catalyzed by a plethora of enzymes. The writers, erasers, and readers are enzymes involved in adding, removing, and recognizing, respectively, these post-transcriptional modifications. The methyltransferases and acetyltransferases act as writers, while the deacetylase and demethylase capable of removing acetyl and methyl groups are classified as erasers. Finally, the readers govern DNA transcription by binding to these modifications.

Altered DNA methylation is mainly related to increased reactive oxygen species (ROS). ROS are generated either directly by iron linked to asbestos fibers or indirectly by inflammatory cells such as the alveolar or peritoneal macrophages acting on asbestos fibers during their passage through the lungs (4). Both ROS-induced mechanisms are involved in methylation and demethylation reactions. ROS induce hypermethylation of gene promoters via a Snail-dependent pathway by recruiting histone deacethylase 1 (HDAC1) and DNA methyl transferase 1 (DNMT1), linking DNA methylation and histone modification (5). Alternatively, ROS oxidize 5-methylcytosine to produce 5-hydroxymethylcytosine (6). This modification, mediated by the ten-eleven translocation methylcytosine dioxygenase (TET) family enzymes, is involved in the process of active demethylation of 5-hydroxymethylcytosine and is responsible for enhancing the transcriptional activity (7).

Changes in DNA methylation and histone modifications lead to silencing of tumor suppressor genes and genomic instability. It is well-established that small non-coding RNAs (miRNAs) are epigenetic modulators and that they themselves can be modulated by epigenetic changes. Alterations of miRNA expression have been reported to link exposure to environmental toxic agents with their pathological consequences, including cancer onset and progression. Asbestos exposure induces early changes in the miRNA machinery; therefore, altered miRNA levels can be proposed as biomarkers of early biological effects. The epigenome is dynamic as well as reversible. Reversible miRNA alterations represent an adaptive mechanism to environmental exposure, while the irreversibility reflects the inability of the cells to restore the physiological miRNA level despite the cessation of exposure to carcinogens. Therefore, it is plausible that miRNA dysregulation induced by carcinogens is predictive of cancer development only when these miRNA alterations become irreversible (8). The change from reversibility to irreversibility of miRNA alterations depends on the duration of the exposure. Long-term asbestos exposure is required to induce asbestos-related malignancies, and the persistence of fibers in the pleura may induce irreversible loss of miRNA function as a result of homozygous deletions of miRNA genes. In addition, the exposure dose also affects miRNA alterations, indicating that early miRNA alterations are affected by both the intensity and the duration of the exposure. While various miRNAs have been found to be deregulated in MM, few miRNAs have been described to be regulated by an epigenetic mechanism. Here, we have reviewed the epigenetic mechanisms involved in miRNA expression in MM, focusing on their role as biomarkers of early diagnosis and on their therapeutic effects.

## Epigenetic Regulation in Malignant Mesothelioma

**Figure 1:**
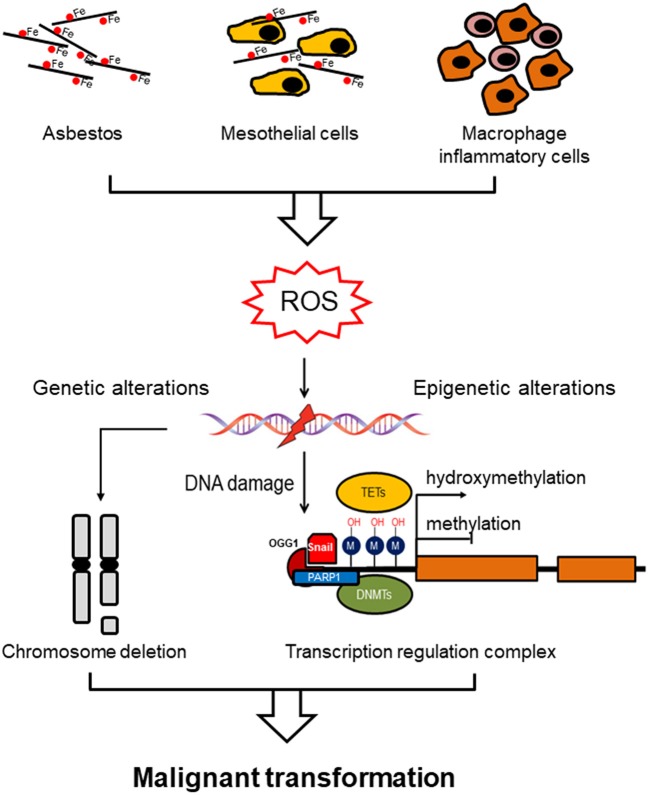
Asbestos induces genomic/epigenomic alterations driving malignant mesothelioma. Asbestos exposure induces ROS formation directly via the iron-induced Fenton reaction or indirectly by chronic inflammation (mesothelial cells and macrophages inflammatory cells). ROS exposure induces methylation of the gene promoter via a specific recognition site to which DNMT1 and PARP1 are recruited, linking DNA damage and DNA methylation. Alternatively, prolonged ROS exposure induces demethylation by oxidizing the 5-methycytosine to produce 5-hydroxymethylcytosine, which is catalyzed by ten-eleven translocation methylcytosine dioxygenase (TET) family of enzymes. Hypomethylation of genomic DNA is associated with genomic instability, which in combination with genetic alterations (chromosome deletion), both contribute to malignant transformation.

All forms of asbestos induce carcinogenicity involving both direct (oxidative stress) and indirect (chronic inflammation) mechanisms. Oxidative stress induced by free radical species (ROS) is considered to be one of the trigger for asbestos-induced pathogenesis. ROS as DNA-damaging agents increase mutation rates and promote malignant transformation, and they also act as signal mediators in redox signaling, which has an impact on several signaling pathways (9). These changes entail DNA oxidation events, post-translational modifications of histones proteins, and DNA methylation. The etiology of MM is associated with genomic mutations but also epigenetic errors leading to dysregulation of gene expression (Figure 1).

### Histone Modifications

DNA is condensed in a complex represented by chromatin, and it is comprised of histones and non-histone proteins. The histone family includes H1, H2A, H2B, H3, and H4, whose covalent modifications have important roles in regulating chromatin dynamics and gene expression. For gene transcription to occur, DNA must be accessible to transcription factors and/or enhancers. The phosphorylation of histones endows them with reduced affinity for DNA, which may contribute to chromatin decondensation, thus allowing for the access of proteins needed for transcription. Phosphorylation of histones has been reported to be linked to DNA damage and ligation.

The acetylation of histones contributes to the expression of genes through transcription activation by changing condensed chromatin into a more relaxed structure, thereby recruiting components of the transcriptional machinery. This post-translational modification is catalyzed by acetyltransferases and deacetylases by means of the acetylation/deacylation of lysine residues. There are three major families of histone acetyltransferases (HATs) and two families of lysine deacetylases, the Zn^2+^-dependent histone deacetylases (HDACs) and the nicotinamide adenine dinucleotide (NAD^+^)-dependent sirtuins (10, 11).

Generally, the acetylation of histones induces transcription activation, while histone methylation, catalyzed by Ezh2 (the catalytic activity of the PRC2 complex), can promote either activation or repression, based on the targeted residue within a particular histone (12). Methylation is a reversible process that is enzymatically regulated; methyl groups can be removed by demethylases of the JMJD2 subfamily, which selectively remove methyl groups from histone 3 lysine 9 (H3K9) (13). The removal of methyl groups is accomplished by successive oxidations of methylated cytosine by specific DNA hydroxylase enzymes in conjunction with the DNA base excision repair machinery. Oxidative stress induced by asbestos exposure transiently alters the epigenetic programmed process by affecting the activity of enzymes responsible for the demethylation and deacetylation of histones (14). ROS catalyzed via Fenton reaction in the presence of Fe (II) linked to the presence of asbestos fibers can increase histone methylation that may be attributed to the inhibition of histone demethylase activity, as previously reported (15, 16).

Previous studies have shown that Poly(ADP-ribose) polymerase-1 (PARP1) is involved in asbestos-induced DNA damage and repair (17, 18). Asbestos activates PARP1 to repair DNA in mesothelial cells; however, it was proposed that exposure to asbestos inhibits its activity, which results in higher DNA instability, thus causing malignant transformation (19).

PARP1 is responsible for most cellular poly(ADP-ribosyl)ation. PAR generation (PARylation) is another covalent post-translational modification; here, adenosine diphosphate (ADP)-ribose moiety from NAD^+^ is transferred onto specific amino acid residues of acceptor proteins or onto pre-existing protein-linked ADP-ribose units. The PARylation of histones has been reported to decrease their affinity for DNA and to alter chromatin structure, therefore affecting several chromatin-dependent processes. The activity of PARP1 has been shown to be stimulated considerably due to the presence of various activators, including DNA damage (20). The role of PARP1 in remodeling chromatin overlaps with its role in DNA repair. Following DNA damage, PARP1 is rapidly recruited to the sites of damage and catalyzes PARylation, which results in the addition of PAR chains onto itself and a variety of acceptor proteins, including histones. This is a very rapid process; however, PAR generated following stress, which can include metabolic and genotoxic or oncogenic stressors, is rapidly degraded by poly(ADP-ribose) glycohydrolase (PARG), which cleaves the ribose–ribose bonds of PARs. The dynamic PARPs/PARG activity maintains the genomic methylation pattern. PARP1 can also participate in the regulation of DNA methylation by inhibiting the activity of DNA methyltransferases (DNMTs).

### DNA Methylation

DNA methylation involves the covalent addition of methyl groups from cytosine to CpG dinucleotides that are concentrated in CpG islands (CGI). CGI are mainly located at annotated transcription start site (TSS) within gene bodies (intragenic) or between genes (intergenic), which are in a non-methylated state when the corresponding gene is transcriptionally active. The CpGs outside of the TSS that are involved in transcriptional initiation during development are methylated, leading to stable gene silencing. The CGI methylation is not the initiating event in gene silencing, but it acts to block in the silent state (21). More specifically, about 80% of the CpG residues that are not within the CGIs are methylated, while CGIs are, as a rule, free of methylation (22).

DNA methylation is related to the downregulation of gene expression. Silencing of CGI promoters by methylation is mediated by DNMT1, which preferentially target the “hemimethylated” DNA and directs the addition of a methyl group to the 5′ carbon position of the cytosine ring (5mC). DNMT1 recognizes hemimethylated DNA and is responsible for the maintenance of DNA methylation patterns during DNA replication, while DNMT3A and DNMT3B function as *de novo* methyltransferases, which add a methyl group to the previously unmodified DNA (23). Methylated DNA can prevent the binding of a particular transcription factor (TF) to the promoter; DNA methylation can also create binding sites for proteins that specifically recognize methylated DNA. However, several studies reported that methylation status did not correlate with gene expression, and about 37% of genes showed an inverse correlation. A promoter with low CpG density or without CpG in the 5′-UTRs might be subject to transcriptional regulation via DNA methylation, or hypermethylated CpG-containing promoters might be transcriptionally active (24, 25). It has been postulated that methylation may play a permissive role by establishing chromatin structure changes, thus allowing transcriptional factors or histone modifications to regulate gene transcription. Nevertheless, some limitations of the methods used for the detection of DNA Methylation have to be taken in account. The method routinely used to detect DNA methylation in a whole genome or CpG is the DNA immunoprecipitation microarray or sequencing (MeDIP-chip/seq), which utilizes anti-methylcytosine antibodies to immunoprecipitate DNA that contains highly methylated CpG sites. The MeDIP-chip/seq has been widely used for analyses of methylated DNA in the different targets; however, it is considered low coverage due to the limit of CpG containing recognition sites. Another inherent limitation of MeDIP-chip/seq is its lower resolution, which leads to artifacts and misleading results (26).

Accordingly, it was reported that the CpG density in the promoter determined how DNA methylation affected gene expression; high CpG density was often found in promoter regions of genes and was usually unmethylated. Methylation of these CGIs resulted in transcriptional silencing (27). DNA methylation is a highly dynamic process where the DNA demethylation process plays a central role. Active DNA demethylation involves methylcytosine dioxygenase (TET) that converts 5mC to 5-hydroxymethylcytosine (5hmC). The oxidized 5hmC derivatives represent short-lived intermediates in the active demethylation process, and they also serve as stable epigenetic changes that exert distinctive regulatory roles (28).

Asbestos-induced ROS formation may promote global hypomethylation in cells by triggering the expression of TET enzymes, thus avoiding interference of DNMT (29). The global hypomethylation of the CpG residues that do not form CGI was found in cancer tissues, while hypermethylation was observed within promoters, leading to aberrant transcription initiation, and genome instability (22, 30). Although hypomethylation of large genome domains is frequent, it is not clear whether these effects are a primary or secondary effect in cancer. Interestingly, *de novo* methylation may potentially cause gene silencing, which contributes to the initiation of tumorigenesis. Prolonged ROS stress was found to induce methylation of the gene promoter involving Snail, a master regulatory transcription factor regulating organogenesis (5). However, the “primary epimutations” are rare, as most *de novo* methylation events are associated with DNA sequence changes, and these mutations are likely to be the primary genetic trigger in carcinogenesis (31, 32).

## Epigenetically Regulated miRNAs in Malignant Mesothelioma

MicroRNAs (miRNAs) are short double-stranded non-coding RNAs (~22 nucleotides) that regulate gene expression at the post-transcriptional level. MiRNAs are transcribed in the nucleus as multiple stem loop structures (primary miRNAs). The primary miRNAs are processing into pre-miRNAs by the RNase III enzyme DROSHA, and they are then transported to the cytoplasm where a dicer enzyme removes hairpin structure yielding a 21 base pair miRNA duplex. The mature miRNAs are then incorporated into the RNA-induced silencing complex (RISC) comprising a RNA-binding protein (RBP), such as the Argonaute (Ago) protein, and several auxiliary factors. The binding of miRNAs to their targets is mediated by the hybridization of 7–8 nucleotides of the miRNAs to their complementary nucleotides in the 3′-untranslated regions of their targets. The RNA-binding domains allow RBP to specifically target RNAs resulting in translational inhibition or degradation of target mRNAs, thereby inhibiting gene expression. It has been established that one miRNA can bind to more than one species of mRNA target. On the other hand, multiple species of miRNAs can bind to the same mRNA targets and enhance translational inhibition (33).

Similarly to genes coding for proteins, the expression of miRNAs is regulated by both genetic and epigenetic mechanisms. DNA hypomethylation/hypermethylation and histone modifications are involved in the regulation of the expression of miRNA promoters. It has been reported that miRNA gene methylation is one order magnitude more frequent than that of the protein-encoding genes (34). A high proportion of miRNA is embedded in CGIs susceptible to methylation, and they are therefore highly prone to be epigenetically regulated (35). MiRNAs that are located in the tumor-associated genomic regions (36) can play two distinct roles in malignancy, either as oncogenes or as tumor suppressors (37). According to the miRNA database (38), most miRNAs (62%) are intragenic, i.e., located within introns or exons of protein-encoding genes, while 38% are intergenic, i.e., located in the regions between annotated genes. The transcription of intergenic miRNA is independent of coding genes having their own transcription regulatory elements, such as the promoter, the transcriptional start site, and the terminal signals. Conversely, intragenic miRNAs are co-expressed with their host genes, although some miRNAs show no obvious correlation with their host gene (39, 40). A number of intronic miRNAs regulate the expression by their own independent promoters. In addition to this, genetic alterations by asbestos involves methylation silencing, and various genes have been found to be methylated in malignant mesothelioma (41).

The methylation profile differred among the histological types, and the mesothelial sarcomatoid tumors (SMM) featured hypermethylation characterized by elevated levels of 5mC (42). The hypermethylated KAZALD1 gene was found in SMM (43). On the other hand, the loss of BRCA1-associated protein-1 (*BAP1*) was mainly observed in epithelial MM (EMM), showing superior diagnostic accuracy in EMM to that in the other two subtypes (44). *BAP1* is the most commonly mutated gene in MM, and its expression is altered by both genetic and epigenetic mechanisms (45, 46). BAP1 affects gene transcription by post-translational modifications through ubiquitination changes (47). Inactivation of *BAP1* cooperates with the loss of either *CDKN2A/2*B (cyclin-dependent kinase inhibitor 2A/2B) or NF2 (neuriofibromin 2) to drive the development of MM, highlighting its role in cell transformation (48). It has been reported that germline mutations of DNA repair genes, including *BAP1*, predispose asbestos-exposed patients to MM (49, 50). While the involvement of somatic *BAP1* mutations in mesothelial tumorigenesis is well described, its epigenetic role is still controversial (51). By analyzing 22 sporadic MM biopsies, Nasu and colleagues found that *BAP1* promoter methylation was not altered in MM (52). On the other hand, genomic profiling of MM identified recurrent mutations in the epigenetic regulatory gene *BAP1* (47). Rather than through genetic/epigenetic alterations, miRNAs may affect *BAP1* gene expression at a post-transcriptional level. A negative correlation was found between the levels of miR-31 and the *BAP1* protein expression in lung cancer (53). The same authors identified miR-31 as a direct target of the *BAP1* gene. Despite the loss of expression of miR-31 due to the deletion of the miR-31 gene in chromosome 9p21.3, which is a common aberration in aggressive forms of MM (54), an epigenetic mechanism has also been involved (55, 56), and its upregulation was associated with a worse prognosis in MM (57).

Various miRNAs have been found deregulated in MM, and their performance as diagnostic/prognostic markers in biological fluids has been extensively reviewed by Lo Russo et al. (58). Nevertheless, methods for the quantification and the type of samples used limit their clinical application. The use of an RNA high-throughput sequencing system may provide more reliable and reproducible data with higher clinical relevance.

On the other hand, few miRNAs that are epigenetically regulated in MM are described in the literature. The miR-34 family was found downregulated in MM by a mechanism that involves promoter methylation (59–61). Methylated miR-34 can be detected in serum samples, and its degree of methylation in circulating DNA has been associated with the development of MM (62).

Similarly, hypermethylation of the miR-145 and miR-126 promoter regions is responsible for the low levels of the miRNA in both malignant mesothelial tissues and mesothelioma cell lines (63–67). MiR-126 is epigenetically modulated in cancer including MM. MiR-126 is located in chromosome 9 (q34,3) within intron 7 of its host gene epidermal growth factor-like domain-containing protein 7 (*EGFL7*), a member of the epidermal growth factor (EGF)-like protein family. *EGFL7* is highly expressed by and acts on endothelial cells, and, thus, its expression is highest when the endothelium is in an active, proliferating state (68, 69). Both miR-126 and *EGFL7* could facilitate independent, albeit complementary mechanisms to regulate angiogenesis and to maintain vascular integrity (70). MiR-126 promotes vascular endothelial growth factor (VEGF)-mediated signaling and angiogenesis by suppressing the Sprouty-related EVH1 domain-containing protein 1 (SPRED1) and phosphoinositide-3-kinase regulatory subunit 2 (PI3KR2), both of which are involved in the inhibition of mitogen-activated protein kinase (MAPK) and PI3K signaling pathways (71, 72). On the other hand, miR-126 inhibits angiogenesis by the direct targeting of VEGF-A (73, 74). In addition, miR-126 is known to play a crucial role in tumor pathogenesis, where it acts as an oncosuppressor by inhibition of the PI3K/AKT pathway (75, 76). Further, MiR-126 was found to target ADAM9 (disintegrin and metalloproteinase domain-containing protein 9), which is highly expressed in cancer. Re-expression of miR-126 resulted in ADAM9 silencing in pancreatic cancer cells, thereby reducing cellular migration, invasion, and induction of the epithelial marker E-cadherin (77–79).

Several reports have indicated that miR-126 expression was downregulated in cancer tissues compared with non-tumor tissues (67), including MM (80–86), and its restoration impaired cell growth, migration, invasive properties, and tumorigenesis (87–91). Promoter methylation resulted in the silencing of miR-126 in colorectal cancer (92, 93), breast cancer (94), lung cancer (95, 96), esophageal squamous cell carcinoma (97), glioma (98), and MM (66). Hypermethylation of the CGI in *EGFL7* intron 2, which harbors the S2 transcriptional initiation site of *EGFL7* mRNA and miR-126, was found in MM and was found to be a significant prognostic factor associated with poor survival (66).

Activation of miR-126 by the inhibition of DNA methylation and histone deacetylation further confirmed the epigenetic mechanism (65, 92). Although miR-126 and the *EGFL7* S2 were upregulated after treatment with chromatin-modifying drugs, a very low DNA methylation level was found in the promoter region in bladder and prostate tumors (65). A similar scenario was observed in MM where cancer tissue and its adjacent non-malignant (NM) counterpart were analyzed for the methylation status of the *EGFL7* S2 promoter region in relation to miR-126 and *EGFL7* expression. The downregulation of both miR-126 and *EGFL7* found in MM tissue was not related to any methylation changes within the *EGFL7* S2 promoter (Gaetani et al., unpublished data), suggesting that miR-126 expression was regulated by structural changes of chromatin rather than by DNA methylation. On the other hand, high expression of PARP1 and DNMT1 was observed in malignant compared to non-malignant tissue. The expression of *EGFL7* and miR-126 correlated positively with each other and correlated negatively with PARP1 and DNMT1 levels. In an “*in-vitro*” stromal model, MM H28 cells co-cultured with fibroblasts and endothelial cells increased PARP1 expression, leading to miR-126 and *EGFL7* downregulation. Furthermore, knocking down PARP1 in MM H28 cells induced miR-126 and *EGFL7* upregulation, which was associated with increased DNMT1 levels. These results point to the involvement of PARP1 and DNMT1 in miR-126 regulation in MM. Both DNMT1 and PARP1 are known to modulate chromatin structure; PARP1, in particular, associates with promoters of actively transcribed genes and exerts both positive and negative effects on gene transcription.

Our group reported that DNMT1 expression paralleled upregulation of PARP1, thereby supporting the role of PARP1 in protecting the DNMT1 promoter from methylation, as previously reported (99, 100). These findings indicate that increased expression of DNMT1 was responsible for the methylation of the *EGFL7* promoter and the ensuing downregulation of miR-126. Aberrantly upregulated DNMT1 and the downregulation of miR-126 associated with promoter hypermethylation of its host gene *EGFL7* were observed in esophageal squamous cell carcinoma (ESCC). Based on these findings, a regulatory DNMT1-miR-126 epigenetic circuit was proposed (97). However, we showed that the methylation status of the *EGFL7* promoter cannot fully explain the modulation of miR-126 expression. This is supported by the notion that DNA methylation does not work alone, but occurs in the context of other epigenetic modifications, such as histone modifications, which constitute epigenetic regulatory miRNA expression.

**Figure 2:**
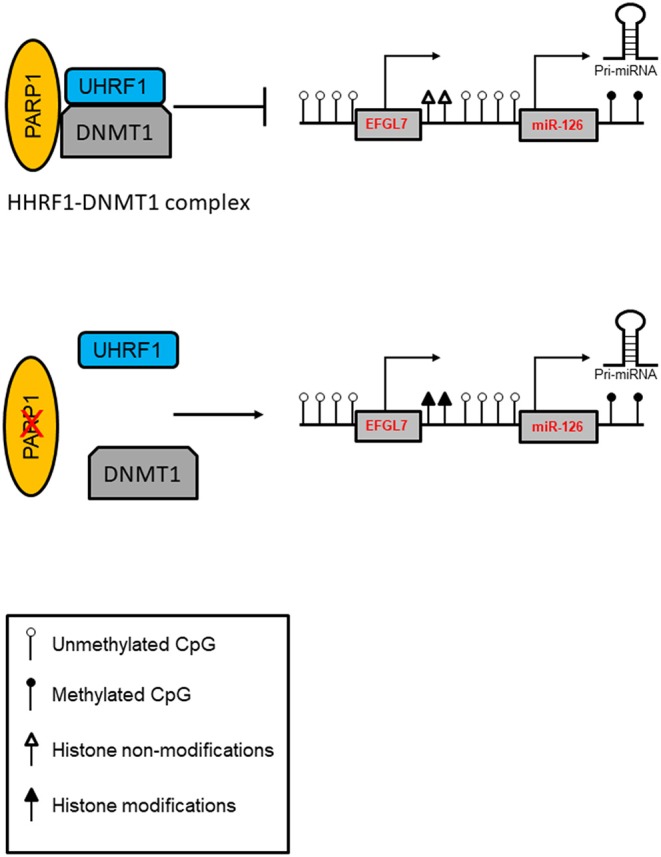
Model of PARP1-mediated control of chromatin structure in the regulation of miRNA expression. PARP1 promotes the interaction of UHRF1 with DNMT1 in regulating miRNA expression independently of DNA methylation. In addition, PARP1 activation facilitates the demethylation of DNA by the recruitment of TET1 and by the exclusion of DNMT1. Inhibition of PARP1 reduces the interaction of the UHRF1-DNMT1 complex that regulates chromatin condensation and enhances transcription.

Intriguingly, asbestos-exposed subjects showed a high miR-126 level (86), and bronchial epithelial cells (BEAS-2B) exposed to asbestos showed increased expression of miR-126 and its host *EGFL7* gene, which was associated with increased expression of DNMT1, and reduced expression of PARP1 in these cells. Notably, both under normal and pathological conditions, the lack of PARP1 resulted in increased DNMT1 expression and consequent miR-126 upregulation. Aberrant promoter methylation was found to contribute to the regulation of this gene. Evidence suggests that the repressive activity of DNMT1 may not be dependent only on DNA methylation, suggesting a “scaffolding” role for the protein to recruit other transcriptional repressive components (101). In this context, PARP1 is a part of a protein complex containing UHRF1 (ubiquitin-like, with PHD and RING finger domains 1), an epigenetic coordinator, and DNMT1 in which PARP1 regulates UHRF1-associated biological activities. A reduced UHRF1-DNMT1 complex was observed in the absence of PARP1, and it did not significantly perturb the catalytic activity of DNMT1. However, PARP1 participates in the UHRF1-mediated chromatin modifications required for gene silencing (102). A proposed model of the PARP1-UHRF1-DNMT1 complex in the regulation of miRNA expression is shown in Figure 2.

Alternatively, the TET family of proteins expressed in response to asbestos-induced oxidative stress may promote active demethylation of the *EGFL7* promoter, thereby contributing to miR-126 accumulation. DNA methyltransferases (DNMT1, DNMT3A, and DNMT3B) in combination with TET proteins that catalyze demethylation have been previously reported to regulate stress-induced miR-126 expression (103).

However, rather than methylation, PARP1 may orchestrate the expression of miR-126 by its upregulation of miR-126 in asbestos-exposed cells, while it may also downregulate miR-126 in MM cells. High PARP1 expression has been observed both in asbestos-exposed subjects, and this is most probably a consequence of oxidative stress induced by asbestos. However, the PARP1 expression did not correlated with its activity. It has been proposed that exposure to asbestos inhibits the PARP1 activity, possibly resulting in higher DNA instability that can lead to malignant transformation (19).

miRNAs can be epigenetically regulated by DNA methylation and/or histone modifications. In turn, certain miRNAs directly target enzymatic effectors involved in epigenetic modulations (104), thus suggesting a regulatory circuit between epigenetic modulation and miRNAs, which could have a significant effect on transcription (95, 105). In ESCC, the overexpression of DNMT1 induced promoter hypermethylation of the miR-126 host gene, which resulted in decreased levels of miR-126. On the other hand, DNMT1 was suppressed by miR-126 overexpression (97). Similarly, it was found that members of the miR-29c family had a direct effect on DNMTs in MM (106), and it was demonstrated that these miRNAs also affected the demethylation pathway (107).

## miRNA-induced Metabolic Changes Affect Epigenetic Regulation in Malignant Mesothelioma

miRNAs are known to regulate epigenetics by affecting various metabolic processes either directly or indirectly (108, 109). The chromatin undergoes continuous modifications, which are dependent on intermediate metabolites, including acetyl-CoA, ATP, NAD^+^, flavin adenine dinucleotide (FAD), α-ketoglutarate (α-KG), and uridine diphosphate (UDP). These metabolic intermediates can serve as cofactors or inhibitors of the enzymatic activity of chromatin modifiers, thereby coupling the chromatin structure with the metabolic state of the cell. For instance, IRS1, an adaptor protein mediating IGF-I/insulin signaling, which is involved in various pathological processes, is a target of miR-126 (90, 110). IRS1 integrates signaling from insulin receptors, insulin-like growth factor-1 receptor (IGF-1R), and many other cytokine receptors, leading to the activation of the PI3K-AKT. AKT promotes the shunting of mitochondrial citrate from the tricarboxylic acid (TCA) cycle to acetyl-CoA production by activation of ATP citrate lyase (ACLY). Acetyl-CoA forms are the universal substrate for the acetylation of histones. The histone acetylation is one of the best-characterized post-translational modifications. The HAT activity, which relies on intracellular levels of acetyl-CoA, connects metabolism to transcriptional regulation by chromatin dynamics. The high activity of pathways resulting in the formation of acetyl-CoA precursors, such as the IRS1-activating IGF-I/insulin signaling, is linked to histone hyperacetylation, which in turn promotes gene expression that modulates cell growth under these “favorable” conditions. Restoration of miR-126 in MM suppressed the IRS1/IGF/AKT pathway and inhibited ACLY, thus contributing to the decrease of acetyl-CoA-mediated histone acetylation (91).

MiR-126 reduced mitochondrial respiration in MM cells and induced the mitochondrial redox activity (MRA) as a result of increased intracellular reductants such as FMNH_2_, FADH_2_, and NADH/NADPH (90). These reducing elements are produced by aerobic glycolysis, and their consumption in the mitochondrial matrix is a consequence of altered homeostasis. The flux through glycolysis determines the NAD^+^/NADH ratio, which is important for the deacetylation activity of sirtuins (SIRTs). Another NAD^+^-dependent protein involved in chromatin remodeling is PARP1. The epigenetic alteration by PARP1 includes the maintenance of H3K4me3 in the trimethylated form, a marker of permissive chromatin, resulting in the inhibition of the histone demethylase and histone deacetylase KDM5B and HDAC. This prevents the aberrant hypermethylation of CGI in the housekeeping gene promoters by DFNMT3a/b (111).

**Figure 3:**
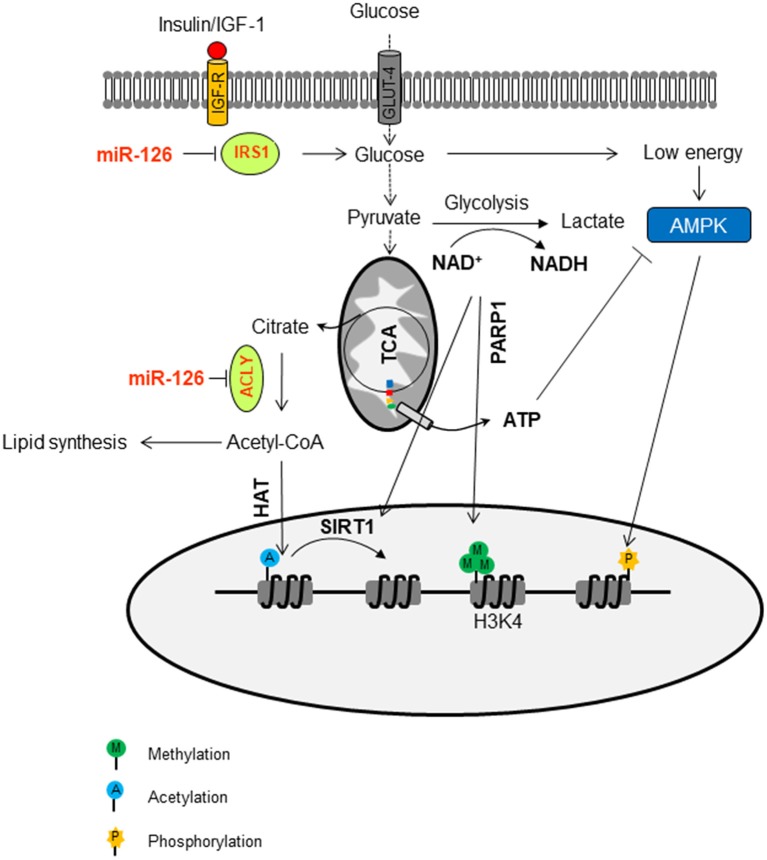
Epigenetic regulation of miR-126 by metabolic changes in malignant mesothelioma. MiR-126 suppresses IRS1 by binding to its 3′-UTR, with ensuing inhibition of the insulin/IGF-1/AKT pathway. This contributes to the change of glucose metabolism. The flux trough glycolysis determines the NAD^+^/NADH ratio, which is involved in the activation of sirtuin histone deacetylases (SIRTs). The NAD^+^-dependent PARP1 is involved in the maintenance of H3K4me3 in the trimethylation form, a marker of permissive chromatin. The ATP/AMP ratio can activate AMPK, a kinase that can phosphorylate histones. In addition, miR-126 inhibits ATP citrate lyase (ACLY), thus increasing the cytosolic citrate, which is converted to acetyl-CoA and used as a donor for histone acetyltransferases (HAT)-mediated histone acetylation.

Intracellular ATP can be involved in the epigenetic regulation as well. The ATP level is above the K_m_ value for most kinases that, in most cases, do have a direct effect on histone phosphorylation. However, some kinases, such as AMPK, which is activated by high AMP/ATP ratios that, in turn, are indicative of metabolic stress, can translocate to the nucleus and specifically phosphorylate histone H2B on serine 36. AMPK-dependent H2B Ser36 phosphorylation is essential for transcription and survival in response to metabolic stress (112). The epigenetic regulation of miR-126 by metabolic changes in MM is documented in Figure 3.

It was proposed that environmental exposure affects the global epigenetic pattern by interfering with the metabolism by a mechanism that involves oxidative stress. Cancer metabolic rewiring could affect the availability of cofactors required for epigenetic changes and generate oncometabolites that act to modify the expression of epigenetic enzymes. On the other hand, epigenetic alteration modifies metabolism by affecting the expression of the relevant enzymes.

## Epigenetic miRNAs as Biomarkers of Malignant Mesothelioma

Epigenetic modifications are observed in early stage tumors; therefore, the detection of epigenetic miRNAs (epi-miRNAs) could be used as an epigenetic biomarker for early detection of cancer. Several microRNA signatures (miRscore) have been performed to identify candidate miRNAs with potential diagnostic and prognostic biomarkers (58, 113, 114). Table 1 summarizes the most specific miRNAs reported by different groups (115, 117–121, 123–125).

**Table 1:** MiRNAs with diagnostic and prognostic value and their targets in malignant mesothelioma.

		**miRNA**	**Target genes**	**Cell function**	**References**
**DIAGNOSIS**		miR-16	Bcl-2, CCND1	Apoptosis, cell cycle	(115, 116)
		miR-103	ICOS, SERBP1, FBXW11	Transcription, genome integrity	(117)
		miR-106	Unknown	–	(118)
		miR-223	PARP1, MDM2, TP53, JNK signaling, STMN1	Cell motility, tubulin acetylation	(119)
		miR-625	Unknown	–	(120)
**PROGNOSIS-DIAGNOSIS**		miR-17	KCNMA1	Cell migration	(115, 121)
		miR-193a	MCL1, PD-L1, E2F1, SRSF2, TYMS	Proliferation, apoptotic/necrotic	(122)
		miR-143	DNMT3A, FOSL2	Proliferation, methylation	(82)
		miR-652	Unknown	–	(82)
		miR-23a	Unknown	–	(113)
		miR-31	PPP6C	Proliferation, migration, invasion, colony formation	(55–57, 113)
	**Epi-miRNA**	miR-34	c-MYC, c-MET, BCL-2, CDKN2, NF2, TP53	Proliferation, invasion, migration apoptosis, differentiation	(59–62)
		miR-145	OCT4, ZEB1	Proliferation, invasion, migration, angiogenesis	(63, 64, 82)
		miR-126	CRK, PI3K/Akt, p85β, IRS1, ADAM9, VEGF, VCAM1, EGFL7, SOX-2	Proliferation, invasion, migration, angiogenesis	(65, 82, 90)
**PROGNOSIS**		miR-29c	DNMT1, DNMT3A	Proliferation, migration, invasion, colony formation, methylation	(106, 107)
		miR-21	PARP1, MSLN	DNA repair	(113, 123, 124)
		miR-30	P53	Tumor suppressor, cell cycle	(113)
		miR-221/222	PTEN, TIMP3, p27Kip1, p57, Bim	Cell invasion, metastasis	(113, 125)

*CCND1, cyclin D1-encoding gene; ICOS, inducible T-cell co-stimulator; SERBP1, SERPINE1 MRNA Binding Protein 1; FBXW11, F-box and WD repeat domain containing 1; PARP1, Poly(ADP-ribose) polymerase-1; MDM2, Mouse double minute 2 homolog; STMN1, Stathmin 1; KCNMA1, calcium-activated potassium channel subunit alpha 1; PD-L1, Programmed death-ligand 1; E2F1, E2F Transcription Factor 1; SRSF2, Splicing factor, arginine/serine-rich 2; TYMS, Thymidylate Synthetase; DNMT3A, DNA methyltransferase-3A, FOSL2, Fos-related antigen 2; PPP6C, Protein Phosphatase 6 Catalytic Subunit; CDKN2, cyclin-dependent kinase inhibitor; NF2, neurofibromatosi tipo 2; OCT-4, octamer-binding transcription factor 4; ZEB1, Zinc Finger E-Box Binding Homeobox 1; IRS1, insulin receptor substrate-1; ADAM9, metalloproteinase domain-containing protein 9; VEGF, vascular endothelial growth factor; VCAM1, vascular cell adhesion molecule 1; EGFL7, epidermal growth factor-like domain-containing protein 7; SOX-2, Sex-determining region Y-box 2; MSLN, mesothelin; PTEN, prime time entertainment network; TIMP3, TIMP metallopeptidase Inhibitor 3*.

**Figure 4:**
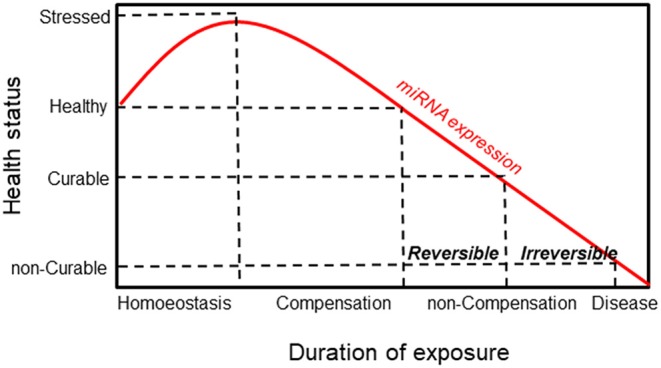
Schematic responses of miRNA and healthy status to environmental exposure. MiRNA expression increases in response to exposure as an adaptive mechanism. This is followed by a compensation phase where miRNA expression is reversible (curable disease). Prolonged exposure induces a non-compensation phase, and the irreversibility of miRNA is associated with the development of the disease (non-curable disease).

Alteration of miRNA expression plays an important pathogenic role in linking carcinogen exposure and its pathological consequences, such as cell transformation. By evaluating the miRNA expression profile in lung cancer tissues and their normal counterparts of highly asbestos-exposed and non-exposed lung cancer patients, 13 deregulated miRNAs that were related to asbestos exposure were identified (126). Recently, Santarelli et al. proposed a panel of four miRNAs (miR-126, miR-205, miR-222, and miR-520g) that were found to be implicated in asbestos-related malignant diseases (86). Notably, increased expression of miR-126 and miR-222 was found in subjects currently exposed to asbestos, such as workers involved in the maintenance and restoration of buildings containing asbestos. Conversely, subjects exposed to asbestos in the past did not show any changes in miRNA expression, suggesting a reversible mechanism. While the reversible miRNA alterations represent an adaptive response to acute carcinogen exposure, long-lasting exposure to carcinogens causes irreversible miRNA alterations that activate carcinogenic mechanisms. Notably, irreversible alterations of miRNA expression can result in cell transformation only when accompanied by other molecular changes. It has been established that the irreversible loss of miRNA in cancer is the result of chromosome deletion or epigenetic-induced silencing of miRNA host genes (127, 128). According to these notions, miRNAs show high sensitivity in detecting exposure to carcinogens and malignancy induced by the exposure itself, representing a general mechanism that links exposure to carcinogens with the pathological consequences (Figure 4).

This is the case for miR-126 in MM, where its expression is modulated in response to asbestos exposure, promoting malignant transformation. MiR-126 expression increases as an adaptive response to asbestos exposure and may proceed to the loss of its expression as a consequence of DNA damage accumulation and chromosome deletion, thus leading to carcinogenesis. Activation of the miRNA machinery is an early event during exposure to carcinogens, thus representing a very sensitive biomarker of their early effect. As reported in Table 2, miR-126 showed an acceptable sensitivity (71 ± 12%) with a low specificity (54 ± 15%) to the differentiation of healthy subjects from patients with MM. However, to complete the carcinogen process, the occurrence of other molecular events is required. Accordingly, multiple biomarkers of different molecular classes should be used to predict cancer development. In this context, the epigenetically regulated miR-126 and miR-145 (epi-miRNAs) were combined with miR-143 and miR-652 in order to differentiate MM from non-neoplastic pleura and reactive mesothelial proliferations (82). Moreover, epi-miR-126 has been associated with specific biomarkers of MM, such as soluble mesothelin-related proteins (SMRPs) (80). When combined with SMRPs, miR-126 indicates a better discriminatory ability to identify tumors in a population of subjects with high risk, suggesting that they have a potential role as predictive biomarkers.

**Table 2:** Ability of miR-126 to distinguish healthy subjects from malignant mesothelioma patients.

**Studies**	**Sample**	**Sensitivity (%)**	**Specificity (%)**	**AUC (area under curve)**
Santarelli et al. (80)	Serum	–	–	0.701 [0.542–0.851], *p* = 0.024
Tomasetti et al. (81)	Serum	70	60	0.894 [0.503–0.968, *p* = 0.0001
Santarelli et al. (83)	Serum	75	54	0.710 [0.568–0.822], *p* = 0.001
Santarelli et al. (86)	Serum	62	30	0.626 [0.524–0.728], *p* = 0.018
Weber et al. (129)	Plasma	59	72	0.614 [0.439–0.789], *p* < 0.05

Indeed, the combination of two circulating epigenetic biomarkers (methylated thrombomodulin and epi-miR-126) with SMRPs for early MM diagnosis overcomes the limitations of using SMRPs alone (83). SMRPs are widely studied tumor markers for the early diagnosis of MM or for monitoring the response to treatment (130). The level of SMRPs can differentiate MM patients from healthy controls with a sensitivity of 60–70% and specificity of 90–100%, and it can better discriminate controls from patients with advanced MM (83, 131). However, this correlation was found only in the 40–50% of epithelioid or biphasic MM histotypes and in the 30% of sarcomatoid MM. A meta-analysis study showed that, in patients suspected of having mesothelioma or high-risk subjects, a negative blood test for SMRPs did not exclude MM even at the high sensitivity threshold (1–1.5 nmol/l) (132). The poor sensitivity of SMRPs clearly limits its clinical value to early diagnosis and emphasizes the need for further biomarkers. The epigenetic biomarkers offer the advantage of increased sensitivity at the expense of specificity, which can be overcome by a combination with specific soluble proteins released directly from the tumor, such as SMRPs, osteopontin (pOPN), vimentin, fibulin-3, and other promising marker for the diagnosis (130).

MM is a highly fatal malignancy featuring rapid development. There are therefore some concerns about the use of biomarkers for its early detection and their impact on the survival of MM patients. The onset of MM is insidious; the diagnosis can be difficult, with clinical symptoms that can mimic many other diseases. Most patients therefore have advanced disease at presentation, which limits the efficacy of current therapies for MM, and the overall prognosis remains poor. The optimal management of MM requires its early diagnosis (133). Although many miRNAs alone or in association with other molecules have been proposed, most of the candidate biomarkers have not been validated in pre-diagnostic samples. In a recent study, three specific miRNAs for MM, including epi-miR-126, were evaluated in pre-diagnostic MM (median of 9 months from the diagnosis). The candidate miRNAs either alone or in combination failed to detect MM, thus it was concluded that they were not feasible for the early detection of this cancer (129). It was reported that downregulation of miRNAs detected in the serum of MM patients was not linked to deregulation of these miRNAs within the tumor (134). It is plausible that the tumor has to reach a certain stage (size) for a specific effect on miRNA expression to be seen.

For instance, the expression of miR-126, which is mainly produced by endothelial cells, is affected not only by cancer but by also by other pathologies involving endothelial dysfunction, including type 2 diabetes and coronary artery disease (67, 135). Thus, the downregulation of circulating miRNAs is a non-specific response to the presence of neoplastic tissue. The diagnostic role of miRNAs is therefore limited, and only when combined with other markers (proteins, miRNA, DNA methylation, and non-coding RNA) may circular miRNA improve their diagnostic performance. While the diagnostic role of miRNAs is questionable, their role in the prediction of prognoses and response to therapy in MM is well established (64, 83).

## Potential Therapeutic Role of epi-miRNAs

Since miRNA can target multiple cell pathways, their use as a therapeutic approach may be important in cancer therapy. Various miRNAs (miR-16, miR-126, miR-145, and miR-193a-3p) and different delivery systems have been tested to inhibit MM tumor growth (63, 90, 116, 122). Both miR-126 and miR-145 are epigenetically regulated, and treatment with the DNA methylation inhibitor 5-aza-2′-deoxycytidine (5-Aza-CdR) restored their expression and, consequently, inhibited cancer growth and invasion (89, 136). The enhanced expression of miR-126 induced the complex metabolic reprogramming of MM, resulting in tumor suppression (90, 91). Similarly, ectopic miR-145 inhibited proliferation, clonal growth, and migration of MM cells, thus reducing *in vivo* tumorigenicity (63). These findings imply that miR-126 and miR-145 have a potential as miRNA-based therapeutic targets for MM.

The only clinical trial (NCT02369198) performed in human MM patients was the phase I, open-label, dose-escalation study. The drug was designed with the aim to restore miR-16, which is frequently downregulated in MM (137). MiR-16 (TargomiR) administrated by the minicell-based formulation (EnGeneIC Dream Vectors) was well-tolerated by MM patients. The observed adverse drug reactions were transient lymphopenia (96%), hypophosphataemia (65%), and increased transaminase levels (23%). Cardiac events (18%) occurred in five cases, including one case of ischemia and one case of Takotsubo cardiomyopathy. In spite of low toxicity, the proportion of patients who achieved an objective response was only 5%, while 68% had stable disease and 27% showed progressive disease (137). A major hurdle in interpreting the data of the phase I study is that TargomiRs rapidly disappear from the circulation after injection. Moreover, immune reactions may occur shortly after the infusion of TargomiRs and may provide an explanation for the antitumor activity observed. The authors concluded that the unmet need of MM patients is very high. On the basis of these Phase I data, a combinatorial therapy seems to be the logical next step in TargomiR development.

Currently, the main barrier to implementing miRNA-based therapy is due to miRNA degradation by nucleases in the circulation and the lack of delivery systems that protect RNAs from nucleases and allow them into the tumor stroma without adverse effects. Exosomes are the physiological carriers of miRNAs, and their involvement in cell-to-cell communication provides an opportunity to deliver therapeutic cargo directly into the cytoplasm of target cells.

## Conclusions

The alteration of miRNA expression is the results of exposure to carcinogens. Unlike chemical carcinogens, the toxicity of asbestos relies in its fibrous nature and persistence, and it involves mechanisms linked to increased ROS production. ROS-induced multiple somatic genetic and epigenetic changes may be required for the tumorigenic conversion of mesothelial cells. The epigenetic modulation of miRNAs occurs early during exposure, representing an adaptive event to defend the cells by activating the detoxifying mechanisms. However, persistent exposure to ROS overwhelms the miRNA-based adaptive response and the irreversible alterations associated with asbestos-induced DNA damage, contributing to cancer development. For example, miR-126 was reversibly expressed following asbestos exposure, while its irreversible downregulation resulted in the activation of the IRS1/PI3K/AKT pathway, which is a frequent event in human cancers as it plays a key role in cancer progression. Hence, its inhibition has become a promising approach to cancer therapy. Ectopic miR-126 inhibited IRS1, thus resulting in metabolic changes and consequent tumor suppression. The miRNA expression links environmental exposure to cancer onset, and this makes miRNAs candidate biomarkers for early detection of MM. Accordingly, the use of miRNAs alone or within a panel of other markers, which includes other miRNAs or molecules (proteins or DNA methylation) has been proposed to predict MM. However, either for the features of the disease or the non-specificity of the candidate miRNAs, the use of these biomarkers for early detection of MM is questionable. Significant changes in miRNA expression were detected only when the MM was manifested. Thus, the characterization of genome-wide DNA epigenetics may offer an opportunity to identify molecules of different classes that may improve the early diagnosis of a fatal type of neoplasia.

## Author Contributions

MT planned and wrote the manuscript. SG and FM acquired the data reported in the manuscript and participated in writing the paragraphs on miRNAs as biomarkers and therapeutic role. JN and LS edited, reviewed, and corrected the manuscript.

### Conflict of Interest

The authors declare that the research was conducted in the absence of any commercial or financial relationships that could be construed as a potential conflict of interest.
